# How Effective Is School-Based Deworming for the Community-Wide Control of Soil-Transmitted Helminths?

**DOI:** 10.1371/journal.pntd.0002027

**Published:** 2013-02-28

**Authors:** Roy M. Anderson, James E. Truscott, Rachel L. Pullan, Simon J. Brooker, T. Deirdre Hollingsworth

**Affiliations:** 1 London Centre for Neglected Tropical Diseases, Department of Infectious Disease Epidemiology, School of Public Health, Imperial College London, London, United Kingdom; 2 Partnership for Child Development, Department of Infectious Disease Epidemiology, School of Public Health, Imperial College London, London, United Kingdom; 3 Faculty of Infectious and Tropical Diseases, London School of Hygiene and Tropical Medicine, London, United Kingdom; 4 Kenya Medical Research Institute–Wellcome Trust Research Programme, Nairobi, Kenya; 5 MRC Centre for Outbreak Analysis and Modelling, Department of Infectious Disease Epidemiology, School of Public Health, Imperial College London, London, United Kingdom; Centers for Disease Control and Prevention, United States of America

## Abstract

**Background:**

The London Declaration on neglected tropical diseases was based in part on a new World Health Organization roadmap to “sustain, expand and extend drug access programmes to ensure the necessary supply of drugs and other interventions to help control by 2020”. Large drug donations from the pharmaceutical industry form the backbone to this aim, especially for soil-transmitted helminths (STHs) raising the question of how best to use these resources. Deworming for STHs is often targeted at school children because they are at greatest risk of morbidity and because it is remarkably cost-effective. However, the impact of school-based deworming on transmission in the wider community remains unclear.

**Methods:**

We first estimate the proportion of parasites targeted by school-based deworming using demography, school enrolment, and data from a small number of example settings where age-specific intensity of infection (either worms or eggs) has been measured for all ages. We also use transmission models to investigate the potential impact of this coverage on transmission for different mixing scenarios.

**Principal Findings:**

In the example settings <30% of the population are 5 to <15 years old. Combining this demography with the infection age-intensity profile we estimate that in one setting school children output as little as 15% of hookworm eggs, whereas in another setting they harbour up to 50% of *Ascaris lumbricoides* worms (the highest proportion of parasites for our examples). In addition, it is estimated that from 40–70% of these children are enrolled at school.

**Conclusions:**

These estimates suggest that, whilst school-based programmes have many important benefits, the proportion of infective stages targeted by school-based deworming may be limited, particularly where hookworm predominates. We discuss the consequences for transmission for a range of scenarios, including when infective stages deposited by children are more likely to contribute to transmission than those from adults.

## Author Summary

Large donations of drugs to treat soil-transmitted helminths (STHs, intestinal worms) means that many more school-aged children will be treated, improving their well-being and development. These children will have to be repeatedly treated since reinfection will occur due to contaminated environments in the absence of improvements in hygiene and sanitation. Repeated treatment of school-aged children may have the added benefit of reductions in levels of infection for the whole community. This will in part be determined by the proportion of the total worms harboured or eggs output by school-aged children, a product of how heavily infected school-aged children are and how many school-aged children there are in the community. In one setting school-aged children output as little as 15% of hookworm eggs whereas in another setting they harbour up to 50% of roundworms. Thus, whilst school-based programmes may have important health benefits, the community-level impact on transmission could be limited unless school-aged children over-contribute to infection. We use mathematical models to show that if children contribute more infective stages to the environment which adults are exposed to than adults do, the reductions in transmission resulting from treating children will be larger, but may still be limited.

## Introduction

In January 2012, a high-level meeting brought together 13 pharmaceutical companies and the global health community in London, UK to announce a new public-private partnership to eliminate or control the seven preventable neglected tropical diseases (NTDs) by 2020, based largely on a new NTD roadmap from the World Health Organization (WHO) [1]. The pledge by pharmaceutical companies to sustain and extend donation programmes facilitates a large portion of the necessary supply of drugs and other interventions to help achieve this goal [2]. Such commitments raise the question of how best to use these resources to induce maximum impact, given that many treatments for NTDs must be administered repeatedly to individuals living in endemic areas due to re-exposure to infection and the absence of fully protective acquired immunity.

The most common NTDs worldwide are the soil-transmitted helminths (STH: *Ascaris lumbricoides, Trichuris trichiura* and the hookworms, *Necator americanus* and *Ancylostoma duodenale*), with an estimated 5.3 billion people worldwide, including 1.0 billion school-aged children, living in areas of stable transmission for at least one STH species [3]. STHs are easily treated with one of four drugs: albendazole and mebendazole, and to a lesser extent, levamisole, and pyrantel pamoate [4]–[5]. However, reinfection commonly occurs [6] due to the inability of the human host to mount protective immunity to reinfection by intestinal helminths [7]–[8], combined with inadequate hygiene and sanitation to restrict or eliminate re-exposure in environments continuously contaminated with the egg or larval free-living transmission stages of these parasitic worms [9].

Following treatment, average worm loads in the population return to their pre-treatment equilibiria in a monotonic manner. The exact dynamics will depend on a number of density-dependent processes that influence parasite reproduction, infection and mortality (in part related to the build-up of a degree of acquired immunity), plus the relatively long life expectancies of established worms in the human host (measured in years) [10]. It will also depend on the proportion of worms in the entire human community in a defined location which are exposed to treatment in a particular control programme.

Deworming programmes for the STHs are often centred on school delivery because of the large burden of morbidity and concomitant developmental consequences for these children [11]–[13], as well as relative ease of access to children in poor rural areas through schools and the cost-effectiveness of school-based deworming [14]–[15]. A number of countries have programmes which additionally include adults, as part of lymphatic filariasis treatment campaigns providing mass treatment with albendazole and ivermectin (or diethylcarbamazine), with associated large impacts on transmission [16]. However, there are large areas where this STHs are not co-endemic for lymphatic filariasis and these areas the WHO-recommended treatment strategies prioritise school-aged children, but also recommend preventive chemotherapy of preschool children, women of childbearing age and adults at high risk [4]. A number of countries are currently implementing only school-based deworming [11]. The large donation of 600 million doses per year announced in the London Declaration almost completely covers the estimated 610 million school-aged children in need of preventive chemotherapy [1]–[2], [11], [17], but it does not cover pre-school children, women of child-bearing age or treatments more than once a year. This paper therefore examines the effectiveness of school-targeted programmes in restricting transmission within the larger community of pre-school children, school-aged children and adults. We use analytical methods deriving from the description of the transmission dynamics of these parasitic worms [18] and demographic plus school attendance information to calculate what fraction of the total population is treated. We also use mathematical models to discuss the impact of school-based programmes on transmission, including scenarios in which infective stages deposited by children are more likely to contribute to transmission than those from adults. We discuss how the impact of a treatment programme could affect the infection dynamics in the population as a whole depending on the, as yet unknown, details of between age-group mixing.

## Methods

The effect of treating school-aged children on the overall transmission dynamics of the parasites depends on a number of factors. These include the fraction of the total worm population harboured by these age groups, the fraction of the eligible school-aged children who attend school and receive treatment, how other age groups are exposed to the eggs or infective larvae produced by school-aged children and vice versa, and drug efficacy. Data exist for the first two factors, which we discuss below. However there is limited evidence to facilitate the exploration of various assumptions such as random or non-random exposure of age groups to the infective stages produced by other age groups in a defined human community. Drug pharmacokinetics and efficacy are also reasonably well documented for the commonly used drugs for STHs and schistosomes (mebendazole [19], albendazole [20]–[22] and praziquantel [23]). In this analysis we estimate the fraction of worms among school-aged children and then discuss the likely impact of this level of coverage on transmission using a suite of infectious disease models. We first outline the parasitological measures and data on which these estimates will be based.

### Epidemiological Measures - Prevalence and Intensity

Monitoring and evaluation of the impact of community-based preventive chemotherapy programmes is based on two epidemiological measures, the prevalence and the intensity of infection. Prevalence represents the fraction or percentage of the population infected and is typically stratified by factors such as age and gender. The intensity of infection or worm burden for STHs is typical measured indirectly by counts of eggs expelled in faeces (eggs per gram of faeces or EPG) and is similarly typically stratified by age and sex. Less commonly, worm burden may be measured by the worms expelled in total faecal output over a defined period post curative chemotherapy [24]–[26]. The two epidemiological measures are statistics of the probability distribution of worm numbers per person. These distributions are typically highly aggregated in form, with the variance exceeding the mean in value. They are well described by the negative binomial probability model [27]. For this distribution the relationship between prevalence 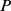 (as a proportion) and mean intensity 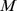 is given by:
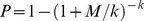
(1)Here, the negative binomial parameter 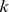 varies inversely with the degree of parasite aggregation within the human population and for values in excess of five the distribution is approximately random in form with the variance approaching the mean in value. Some typical estimates of the magnitude of 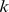 are recorded in Table 1.

**Table 1:** Estimates for the negative binomial parasite aggregation parameter, 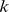.

Parasite	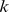 value or range	Distribution measure	Data source
*Ascaris lumbricoides*	0.80	Worm numbers	Elkins et al. (1986) [24]
*Ascaris lumbricoides*	0.81	Worm numbers	Croll et al. (1982) [34]
*Ascaris lumbricoides*	0.44	Worm numbers	Martin et al. (1983) [58]
*Ascaris lumbricoides*	0.46	Worm numbers	Thein-Hlaing et al. (1987) [33]
*Ascaris lumbricoides*	0.36–0.54	Worm numbers	Chai et al. (1985) [59]
*Ascaris lumbricoides*	0.59	Worm numbers	Bundy et al. (1987) [35]
*Necator americanus*	0.34	Eggs per gram of faeces	Bradley et al. (1993) [60]
*Necator americanus*	0.33–0.61	Eggs per gram of faeces	Quinnell et al. (1993) [36], [61]
*Trichuris trichiura*	0.11–0.65	Eggs per gram of faeces	Bundy et al. (1987) [35]

**Small** 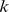 **indicates higher parasite aggregation.**

**Figure 1:**
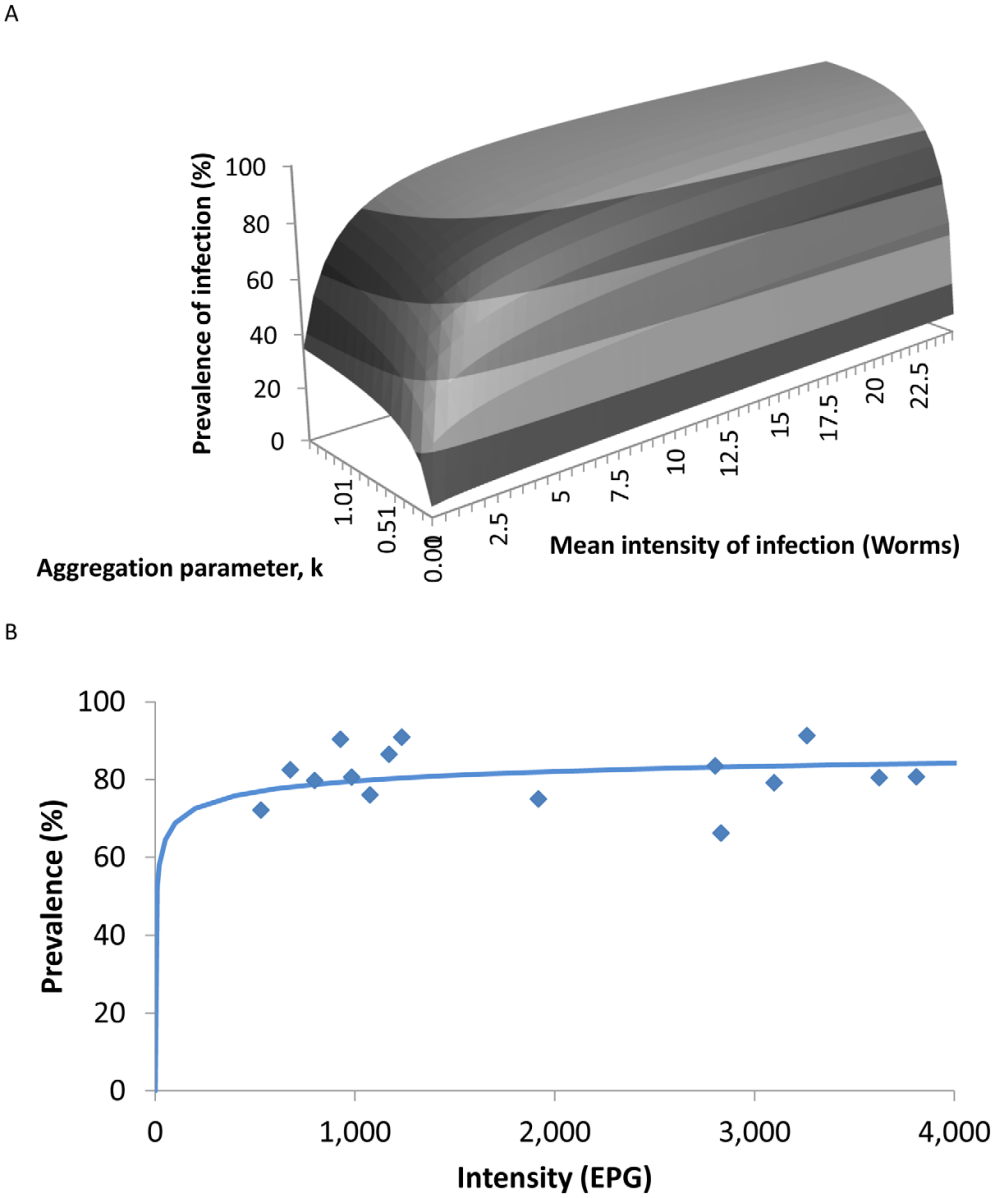
The relationship between mean intensity and prevalence. **A** The relationship between the mean intensity of infection, 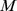, the prevalence of infection, 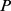, and the negative binomial aggregation parameter, 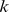 as described by the relationship in equation 1. **B** Relationship between the prevalence and intensity of infection as observed in a study of *A. lumbricoides* [28]. The solid line is the predicted relationship between mean prevalence of infection and worm burden described in equation 1 and plotted in A fitted to estimate the aggregation parameter, 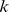 = 0.194.

A plot of the relationship between 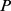 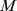 and 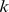 for the negative binomial is presented in Figure 1. It is clear from this figure that the prevalence is a very poor measure of the impact of community-based chemotherapy. Large changes in average worm load as a consequence of treatment will only have a small effect on prevalence unless the mean worm burden is low (i.e. when transmission is very low). For example, in a study in Myanmar two villages with mean EPGs of about 4000 and about 400, an order of magnitude difference in intensity, had almost no difference in prevalence (Figure 1B [28]) and so highly effective treatment of the high intensity village, reducing the burden by a factor of 10, might be viewed as a failed programme if only prevalence were monitored.

The cost and difficulty of monitoring intensity, as opposed to prevalence, is of course greater. However, if monitoring is to have any value, intensity must be measured in some fraction of the treated population. The issue of how best to sample to gain an accurate picture of the impact of treatment, while attempting to keep sample size and the concomitant costs low, requires careful thought. The underlying distribution of parasite numbers per host is central and given its heterogeneity, small sample sizes will not provide robust measures of trends [29]. One compromise is to monitor impact in a subset of age classes – one of children and one in the adult age groups, to see how the treatment of the school-aged children impacts on transmission to adults [30].

### Surveillance and Epidemiological Databases

The greatly expanded deworming programmes seen in many regions of the world in recent years have not been accompanied by systematic recording of treatments delivered and the associated impact on prevalence and intensity. Recently, however, some progress has been made on the generation of open access databases recording global and national spatial distributions of helminths based on estimates of the infection prevalence, as illustrated, by the Global Atlas of Helminth Infection (http://www.thiswormyworld.org [12]) and the Global Neglected Tropical Diseases database (http://www.gntd.org [31]). The Global Atlas of Helminth Infection will be expanded in the near future to include measures of the intensity of infection and treatments delivered. The website will also be extended to encourage the electronic deposition of data on STHs collected in association with the current expanded efforts on community-based control using mass or school-aged targeted anthelminthic treatment. Such data are collected by a number of excellent NTD programmes but is rarely subject to detailed analysis on trends in transmission [32].

The present absence of international databases on treatment of STH and impact of such treatment does make analysis of questions concerning the optimal delivery strategy for community-based programme somewhat challenging. We therefore base our analyses on a small number of available well-designed studies that record prevalence and intensity of infection, stratified by age and sex, before and after various treatment programmes. The age-profiles used are from studies of *A. lumbricoides* in Myanmar [33], India [24] and Iran [34]; *T. trichiura* in St Lucia [35]; hookworm in Uganda [36] and Vietman [37] and, for comparison, studies of *Schistosoma mansoni* in Uganda [38] and Brazil [39].

### Fraction of the Total Worm Population in School-Aged Children (5 to 14 Years of Age)

Epidemiological studies that record the mean intensity of infection stratified by age, when combined with demographic plus school enrolment data, provide information on the fraction of the total worm population exposed to treatment. Along with measures of drug efficacy, this in turn gives the fraction of the total worm population removed by school-aged targeted chemotherapy. The importance of this fraction to the overall transmission dynamics of the target parasites cannot be overstated – and in current control programmes it is an unmeasured parameter.

If 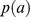 is the proportion of the total population in age class a, the proportion of the human population in the school age classes 5 to 14 years of age, 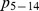 is given by:
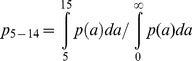
(2)The proportion of the total worm population harboured at time 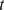 by people between the ages of 5 to 14 years, 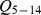, is then given by:
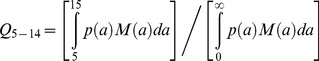
(3)where 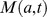 is the mean worm burden in age-group 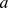 at time 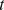.

If the measure of intensity of infection is eggs per gram of faeces (EPG), then the proportion of egg output produced by school-aged children is:
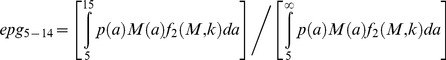
(4)Here 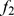 is the density-dependent egg output function, which gives the expected egg output for an individual with mean worm burden 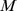 for age 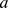 and negative binomial aggregation parameter 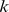. In practice egg output is usually measured, rather than mean worm burden, so we can approximate this by
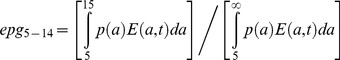
(5)where 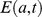 the mean egg output in age-group 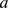 at time 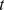. We calculate these fractions for some example datasets on parasite distributions, together with demographic and school enrollment data.

### Demographic Data

Demographic data from various countries where STHs are endemic provides an initial template to assess these issues [40]. Table 2 records the fraction of various populations in the school ages of 5 up to 14 years. In general, within countries where helminth infections are endemic, the fraction of the total population in the school going groups is between 11% and 30%.

**Table 2:** Percentage of population aged 5 to 14 years in 2011 [40].

Country	Percentage of population 5–14 years of age	Total population in millions
Uganda	29.7	33.4
Nigeria	26.9	161.6
Rwanda	26.3	11.1
Kenya	26.3	41.9
Burundi	23.7	8.4
India	19.7	1190.0
Myanmar	18.4	54.0
Thailand	13.4	41.9
People's Republic of China	11.8	1340.0
United Kingdom	11.2	62.7

### School Enrolment Data

**Figure 2:**
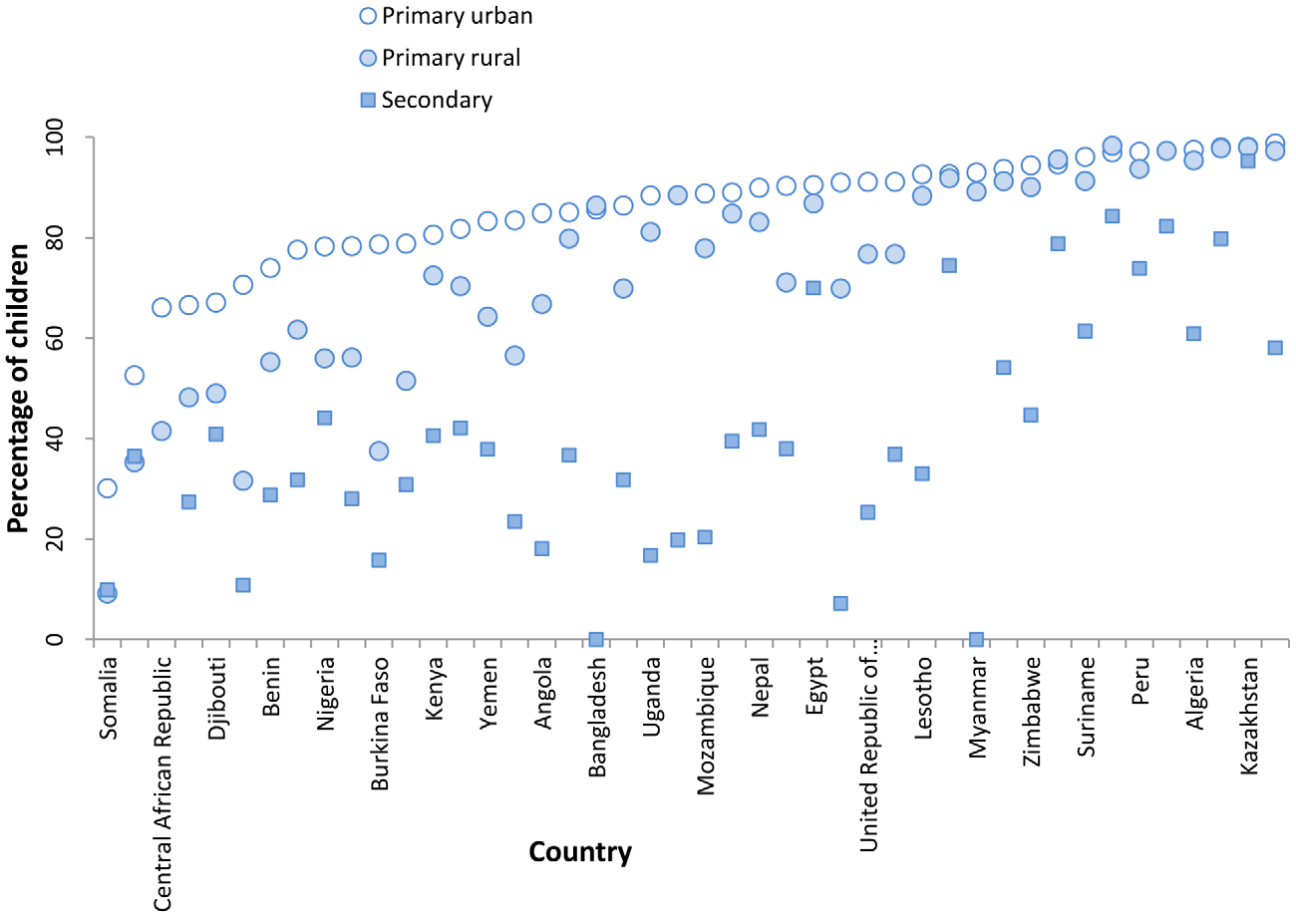
School attendance for a selection of countries. This figure was generated by data published by UNICEF for 2005–2010 [41].. For each country there is net attendance rate at primary, in urban (open circles) and rural areas (closed circles) and a net attendance rate for secondary schools (filled squares).

UNESCO and the World Bank provide data on school enrolment by sex, location (urban or rural) and country. Recent data are recorded in Figure 2 for rural and urban areas for a selection of countries [41] and in Table 6 for enrollement of female students. Over the past decade there has been a steady increase in school enrolment in most countries throughout the world. Progress has been less good in poor rural areas by comparison with urban districts in developing countries. Generally, the most recent data (2005 and beyond) suggest figures in the 80% to 90% range for most urban areas, but with a range of 20% to 60% in some sub-Saharan African countries in rural areas. There is often a gender bias in many poor countries, with attendance figures for females lower than those for males in the primary and secondary school enrolment data. Poor attendance could severely reduce the population-level impact of school-based deworming. Conversely however, there is anecdotal evidence that there may be higher attendance to schools for deworming days due to awareness of the health benefits. These effects have not yet been quantified, to our knowledge.

**Table 6:** Percentage of female children who are enrolled in school [45].

Country	Children enrolled in school, %
Niger	45.5
Eritrea	49.9
Papua New Guinea	50.3
Djibouti	52.1
Chad	60.8
Central African Republic	61.2
Sudan	61.2
Cote d'Ivoire	63.7
Burkina Faso	65.9
Cook Islands	71.3

### Effect of coverage on Programme Impact

For a given coverage of a school-based programme, as defined by proportion of estimated treatment of a proportion of worms , 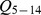, or egg output, 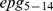, the impact on transmission will depend on the particular dynamics of the parasite. Here we outline general insights on the non-linear effects of limited coverage on transmission. We then use heterogeneous mixing models to investigate how different mixing patterns between adults and children will affect the impact of targeted programmes.

### The Basic Reproductive Number (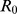) and Parasite Life Expectancy - Their Effects on the Impact of Community-Based Chemotherapy

Simple theory provides some important general insights into the factors controlling helminth transmission and the impact of community-based chemotherapy [18]. For directly transmitted helminths with a free living larval or egg stage outside the human host, the basic dynamics of the system can be described by the following differential equation,
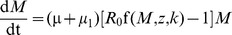
(6)where 1/μ is human life expectancy, 1/μ_1_ is adult parasite life expectancy, *M* is the mean number of worms in the population, *f*(*M*) is the mean egg output per gram of stool, given a mean worm burden of *M*, the dispersal parameter, *k*, and fecundity coefficient, *z*:
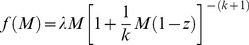
(7)The basic reproductive number defined as the average number of female worm offspring that survive to reproduce in the absence of density-dependent constraints (ignoring the complexities of mating probabilities and age structure in the human population) is given by equation 8

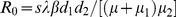
(8)Here, 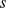 denotes the fraction of female worms, 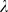 the per capita egg production rate, 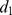 the proportion of female worms that survive in the human host to reproductive maturity, 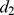 the fraction of eggs or larvae that survive to the infective state, 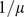 is human life expectancy, 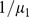 is the adult worm life expectancy in the human host, 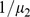 is infective stage life expectancy and 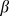 is the per capita transmission coefficient for the infective stage. Table 3 records published estimates of 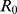 for various helminth species.

**Table 3:** Published estimates of the basic reproductive number 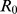 for various helminths.

Parasite	Basic reproductive number, 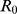	Measure on which parameter estimated	Data source
*Necator americanus*	2.0	Re-infection post treatment	Bradley et al. (1993) [60]
*Ascaris lumbricoides*	4.3	Re-infection post treatment	Croll et al. (1982) [34]
*Ascaris lumbricoides*	1.7	Re-infection post treatment	Thein-Hlaing et al. (1987) [33]
*Trichuris trichiura*	4–6	Re-infection post treatment	Bundy et al. (1987) [35]

A rough approximation of the growth rate 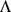 of the parasite population post extensive treatment, again ignoring the effects of density-dependence and worm mating probabilities, is given by:
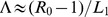
(9)Where 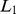 (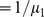) is the parasite life expectancy in the human host. Equation 9 is based on the assumption that 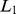 is much shorter than human life expectancy, which is true for all STHs (see Table 4).

**Table 4:** Published estimates of parasite life expectancy, 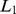, in the human host [18], [27].

Parasite	Life expectancy in years, 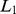
*Enterobius vermicularis*	<1
*Trichuris trichiura*	1–2
*Ascaris lumbricoides*	1–2
*Necator americanus*	2–3
*Ancylostoma duodenale*	2–3
*Schistosoma mansoni*	3–5
*Schistosoma haematobium*	3–5

Ignoring age structure in the human population, the effect of treatment on the effective reproduction number, 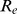, is described by
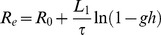
(10)where 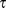 is the interval between successive rounds of treatment and 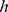 is the efficacy of the drug, defined as the proportion of worms killed by the drug. The critical fraction of the human population that must be treated, 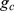, to reduce the effective reproductive number 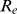 to less than unity in value (assuming that the breakpoint in transmission is close to zero due to the aggregated distribution of worms per person [27]) is given by:
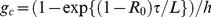
(11)And thus, the fraction 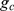 is simply determined by 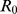, 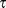, 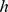 and adult worm life expectancy 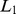. We simulate these approximations for a range of parameter values to illustrate the non-linearities in these relationships.

### Heterogeneity Between Age Classes in Contact with Infective Stages

These approximations for homogenous mixing models are very useful to illustrate the impact of limited coverage on transmission. To gain a more accurate picture of how the treatment of just school-aged children influences the overall transmission dynamics of the parasite, the impact of not only age structure but also exposure to infective stages produced by all age groups in the population must be taken into account. The homogeneous model described above can be extended to an age-structured model to describe the characteristic age-profiles of intensity [18]. Such models usually assume that exposure to infective stages across all age classes is random and independent of which age class produces the eggs or infective larvae. However, the spatial structure of egg deposition and infective stage development arising from one age group, plus their concomitant contact with other age groups, is in reality unlikely to be random in a defined community. Instead, it may be more likely that infective stages produced by school-aged children are deposited in areas closer to habitation, and hence acquired by all age groups of the population, while those arising from adults are less likely to come into contact with children. Therefore the model should include heterogeneous mixing [42].

A simple way to mimic non-random contact is to stratify the population into two age groups, namely; school-aged children (5–14 years), and the rest (0–4 and ≥15 years combined for simplicity, although patterns may also be different between these two groups), and assume different contact patterns with the infective stages within and between these larger age groupings. Such a stratification of hosts has the further advantage of facilitating the modelling of school-based treatment programmes. We assume that the child and adult age groups have negative-binomially distributed worm distributions with the same shape parameter, *k*, but different means, *M_c_* and *M_a_*, respectively. The means evolve independently according to the degree of contact of each group with a common infectious ‘reservoir’. The model equations are (extending the approach by Chan et al. [42])
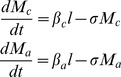
(12)The quantity, 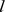, is the per capita infectiousness of the shared reservoir. The parameters 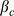 and 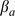 determine the strength of contact with the reservoir for children and adults respectively. The dynamics of the infectious reservoir are described by the following equation
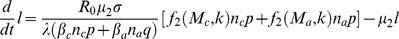
(13)The parameters in equation 13 are as defined earlier. The parameters 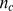 and 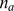 represent the proportion of the population in the two age classes and 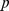 and 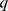 the fraction of egg output that enters the reservoir from children aged 5–14 years and other age groups, respectively.

We investigate the effect of regular school-based treatment on the evolution of worm burdens in the community for three scenarios:

- Homogeneous model. As defined in equation 7. Treatment is applied to a fraction 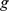 of the population with efficacy 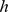 and at intervals of 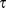 years.
- Heterogeneous model. As described in equations 12 and 13 assuming children and adults are identical epidemiologically. Treatment is applied to a fraction 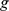 of school-aged children with efficacy 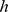 and at intervals of 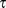 years.
- Heterogeneous model with heterogeneous exposure. As described in equations 12 and 13 assuming that children are both twice as strong a source of eggs and have twice the infectious contact rate. Treatment is applied to a fraction 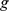 of school-aged children with efficacy 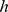 and at intervals of 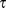 years.

It should be noted that under scenario C there will be higher intensity of parasites in children at equilibrium, or baseline, as a consequence of their higher exposure to infection.We use the mathematical model described above (equations 12 and 13) to simulate how the mean intensity of infection would vary for a setting with a given mean intensity and treatment coverage under each of these different scenarios. We run the mathematical model for *A. lumbricoides* and hookworm, which have contrasting transmission dynamics, to illustrate the importance of age-dependent mixing in the success of an age-targeted treatment programme.

## Results

### Fraction of the Total Worm Population in School-Aged Children (5 to 14 Years of Age)

**Figure 3:**
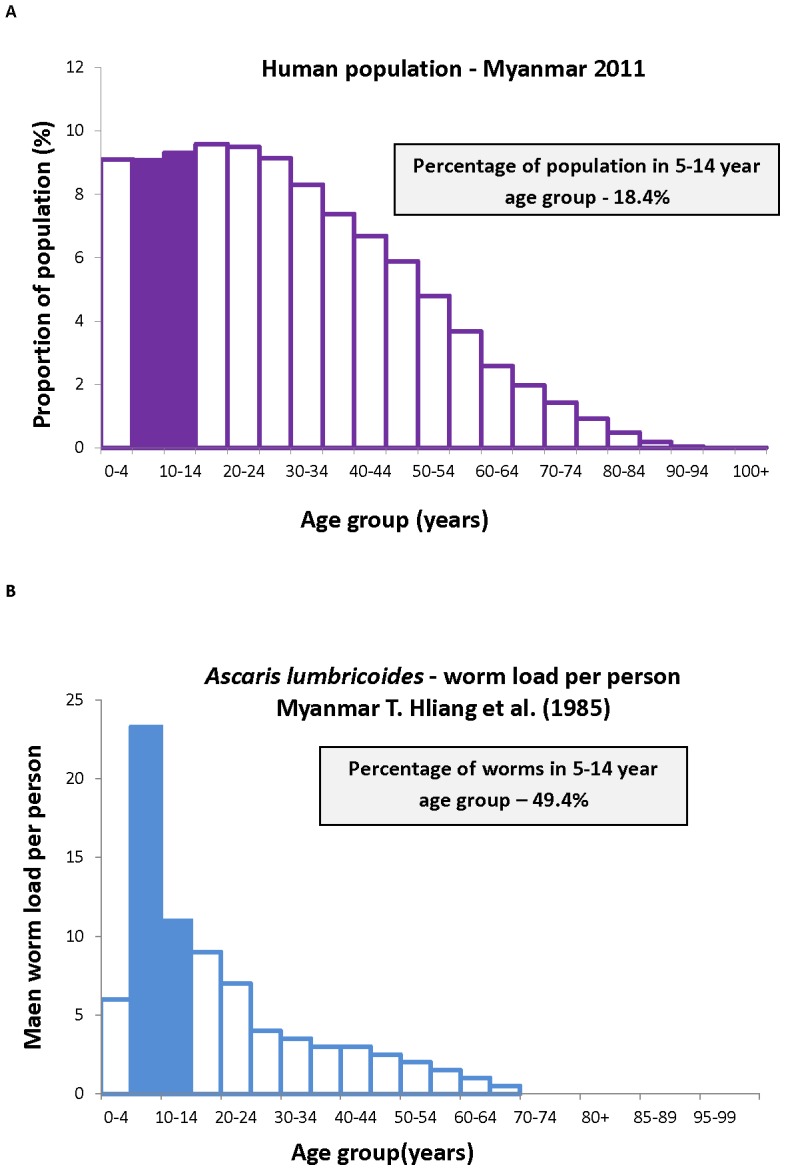
The proportion of *A. lumbricoides* worms in children aged 5–14, calculated from **equation 3**. The demography of the population, A, results in a proportion of 18.4% of the population aged 5–14 years old [45]. Combining this distribution with B the distribution of worms per person by age [57] gives 49.5% of worms in the school-aged group.

**Figure 4:**
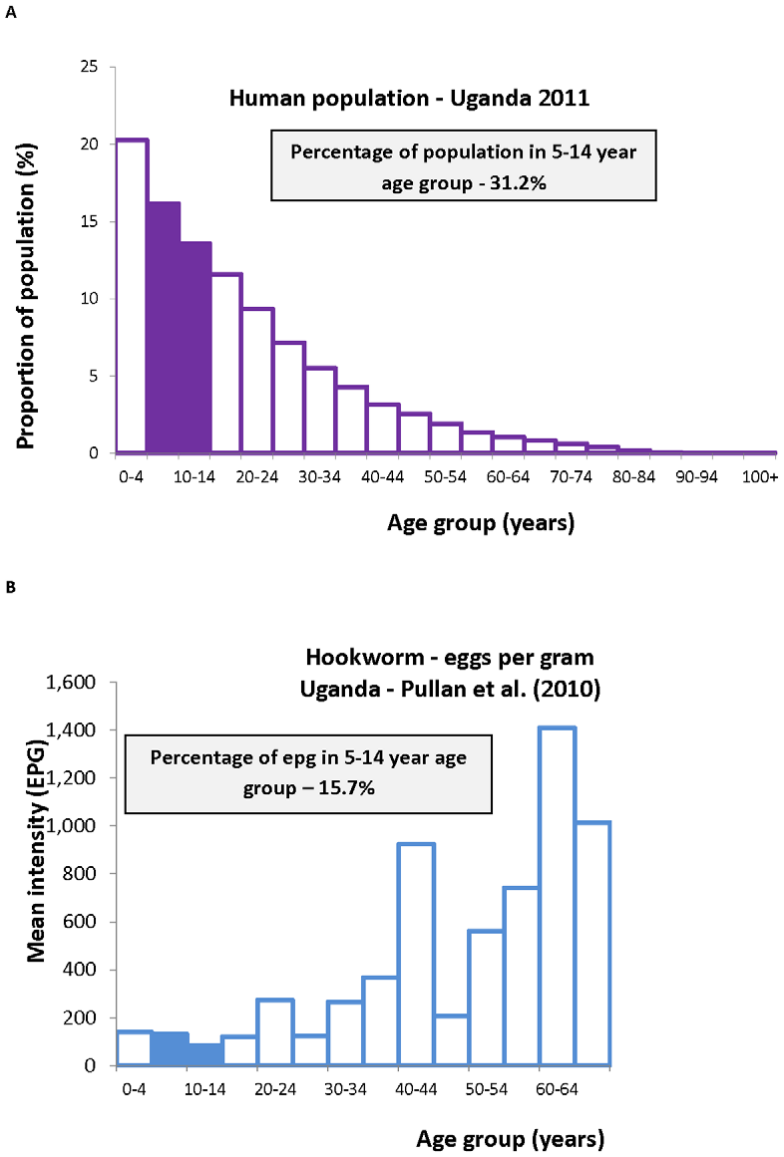
The proportion of hookworm eggs deposited by children aged 5–14, calculated from **equation 5**. The demography of the population, A, gives a proportion of 31.2% of the population aged 5–14 years old [45]. Combining this distribution with B the distribution of egg output by age [36] gives 15.7% of worms in the school-aged group.

The proportion of worms or egg-output in school-aged children, as calculated using equations 3 and 5 above, is best illustrated by reference to a set of published studies covering the main parasites in different country settings. The first example is that of *A. lumbricoides* in Myanmar from a study by Thein-Hliang et al. [33]. The demography and age intensity data are presented in Figure 3. Demographic surveys for the year of the study reveal that treatment of all children in the 5–14 years age range would expose roughly 49.4% of the worm population to anthelminthics. In terms of the fraction of the parasites killed by a school-based programme assuming a drug efficacy of 95% [5] and school attendance of 95% on the days of treatment gives an overall estimate of 44.5% of the total worm population removed by one round of treatment. Calculations for a study by Elkins et al. [24] of *A. lumbricoides* in Tamil Nadu in India yield a very similar figure for the percentage of worms potentially removed by the treatment of children attending school. Calculations for hookworm (largely *N. americanus*) in Uganda from a study by Pullan et al. [36] are significantly different, as shown in Figure 4. For this population only 15% of the egg-output is generated by school-aged children. A drug efficacy of 95% and school attendance figure of 85% in a rural region (see Figure 2), produces an estimate of 12% of egg output treated by one round of treatment. These contrasting figures for *A. lumbricoides* and hookworm illustrate the importance of the shape of the age intensity profile to both the efficiency of school-aged targeting and the degree to which such treatment programmes will impact on overall transmission in a population. Analyses of epidemiological studies of *Schistosoma mansoni* produce figures similar to *A. lumbricoides*, given marked convexity in the intensity by age profiles (age intensity data from Kabatereine et al. [38]). *T. trichiura* is somewhat intermediary between *A. lumbricoides* and hookworm with a degree of convexity but significant worm loads in adults [35], [43]–[44]. The more convex the age intensity profile is, the greater the impact of school-based deworming on overall transmission, provided the peak intensity lies within the age ranges 5–14 years. The continued rise of hookworm intensity in adult age classes yields a low fraction of the total worms in the treated classes.

The calculations presented in Figures 3 and 4 and Table 5 are approximations to the values defined in equations 3 and 5, based on the assumptions that human population size is constant but with an age distribution as documented by the US Bureau of Population and [40], and that the effects of density-dependent fecundity (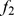) are negligible. Polynomial fits are employed to calculate integrals of the functions 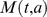 and 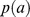 respectively from cross sectional studies of the intensity of infection, and age pyramids (both sexes combined) from national demographic data. The calculations are conservative estimates since they are based on the assumption that all who are eligible attend school. In practice, the percentages recorded in Table 5 must be multiplied by the school attendance data from recent UNESCO studies [45].

**Table 5:** Fraction of worm population or egg output in 5–14 year olds (equations 3 and 5).

Parasite	Percentage of the total population of parasite or egg output in the 5–14 year old age groups	Measure of parasite load	Data source	Country
*Ascaris lumbricoides*	49.4	Worm numbers	Thein Hliang et al. (1987) [33]	Myanmar
*Ascaris lumbricoides*	33.2	Worm numbers	Elkins et al. (1986) [24]	India
*Ascaris lumbricoides*	27.6	Worm numbers	Croll et al. (1982) [34]	Iran
*Trichuris trichiura*	34.1	Eggs per gram of faeces	Bundy et al. (1987) [35]	St Lucia
Hookworm (*Necator americanus*)	15.7	Eggs per gram of faeces	Pullan et al. (2010) [36]	Uganda
Hookworm (*Necator americanus*)	9.4	Eggs per gram of faeces	Needham et al. (1998) [37]	Vietnam
*Schistosoma mansoni*	39.7	Eggs per gram of faeces	Kabatereine et al. (2004) [38]	Uganda
*Schistosoma mansoni*	27.4	Eggs per gram of faeces	Brooker et al. (2007) [39]	Brazil

### The Basic Reproductive Number (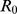) and Parasite Life Expectancy - Their Effects on the Impact of Community-Based Chemotherapy

**Figure 5:**
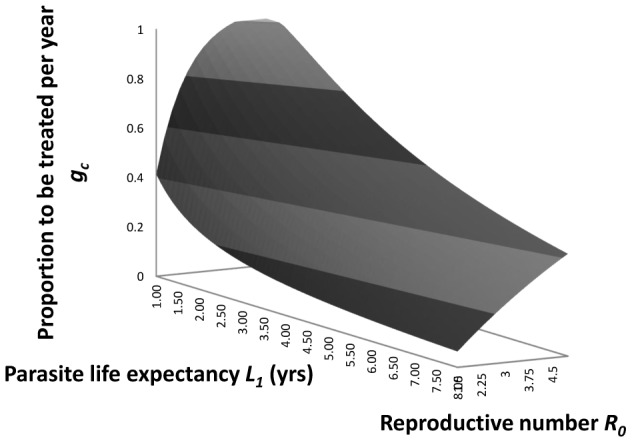
Critical fraction of the population to be treated. The predicted relationship between the critical fraction of the human population to be treated, 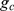, per annum with efficacy, 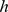, 0.9, and the basic reproductive number, 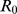, and parasite life expectancy, 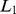 in years (from equation 11 in the main text).

**Figure 6:**
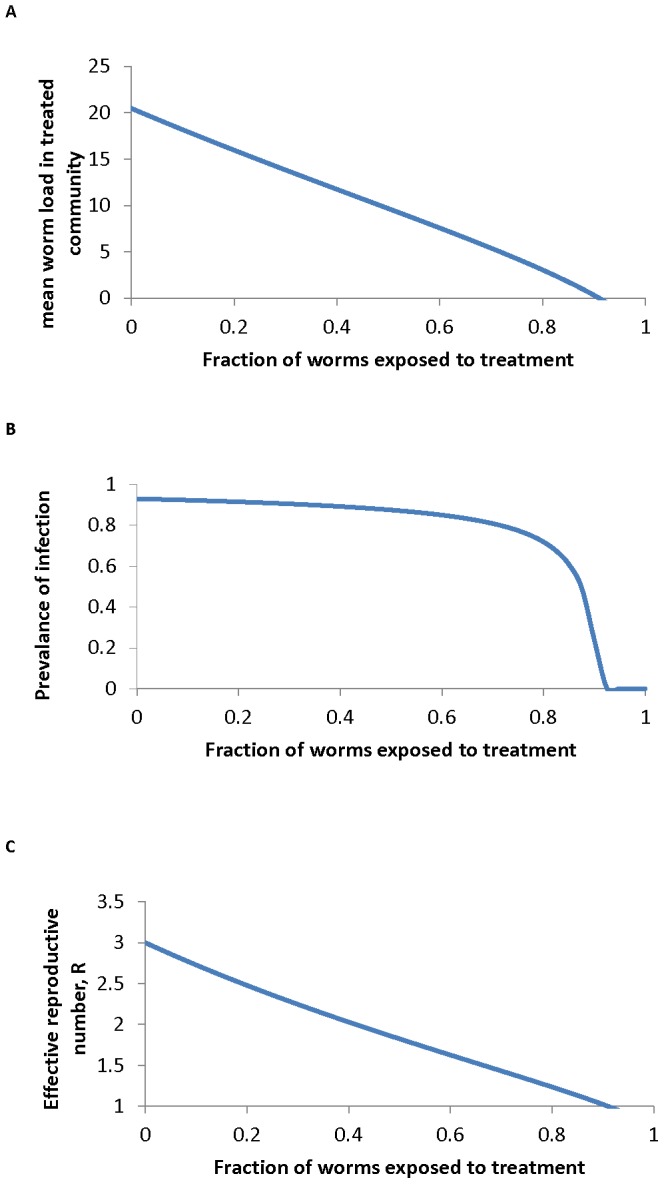
Impact of fraction treated on worm burden, prevalence and effective reproduction number. The impact of the fraction of the population treated, 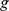, on A the mean worm burden 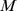, B the prevalence of infection, 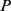 and C the effective reproductive number 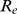, as described in equation 10. Parameter values are set for *A. lumbricoides* as follows: 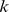 = 0.81, 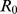 = 3, 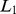 = 1 yr, 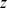 = 0.967 and 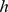 = 0.95.

The impact of the parasite coverage levels presented in Table 5 on transmission under the assumption of homogeneous mixing can be seen using the approximations to the critical treatment coverage, 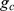 (derived above in relation to the parasite life expectancy, 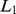 , and the basic reproduction number, 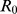, as in Figure 5 (equation 11)) and the impact of increasing 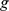 on the mean worm load, 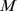, and the prevalence of infection, 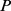 (Figure 6, equation 6). Figure 6 reveals that the mean worm load 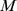 decays approximately linearly as the fraction treated rises, while prevalence 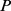 only begins to fall steeply as the fraction treated approaches the critical value 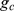 and effective transmission ceases. Note that from Figure 5 the critical fraction treated reaches 1 for high 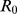/low parasite life expectancy. This indicates the parasite may be very difficult to eradicate by treatment with the given efficacy (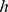 = 0.9) when 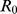 is large (∼>4, estimated values for particular parasites in Table 3) and 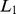 is short (∼<1.2 years, values in Table 4) e.g.for *A. lumbricoides and T. trichiura* in high transmission settings.

### Heterogeneity Between Age Classes in Contact with Infective Stages

**Figure 7:**
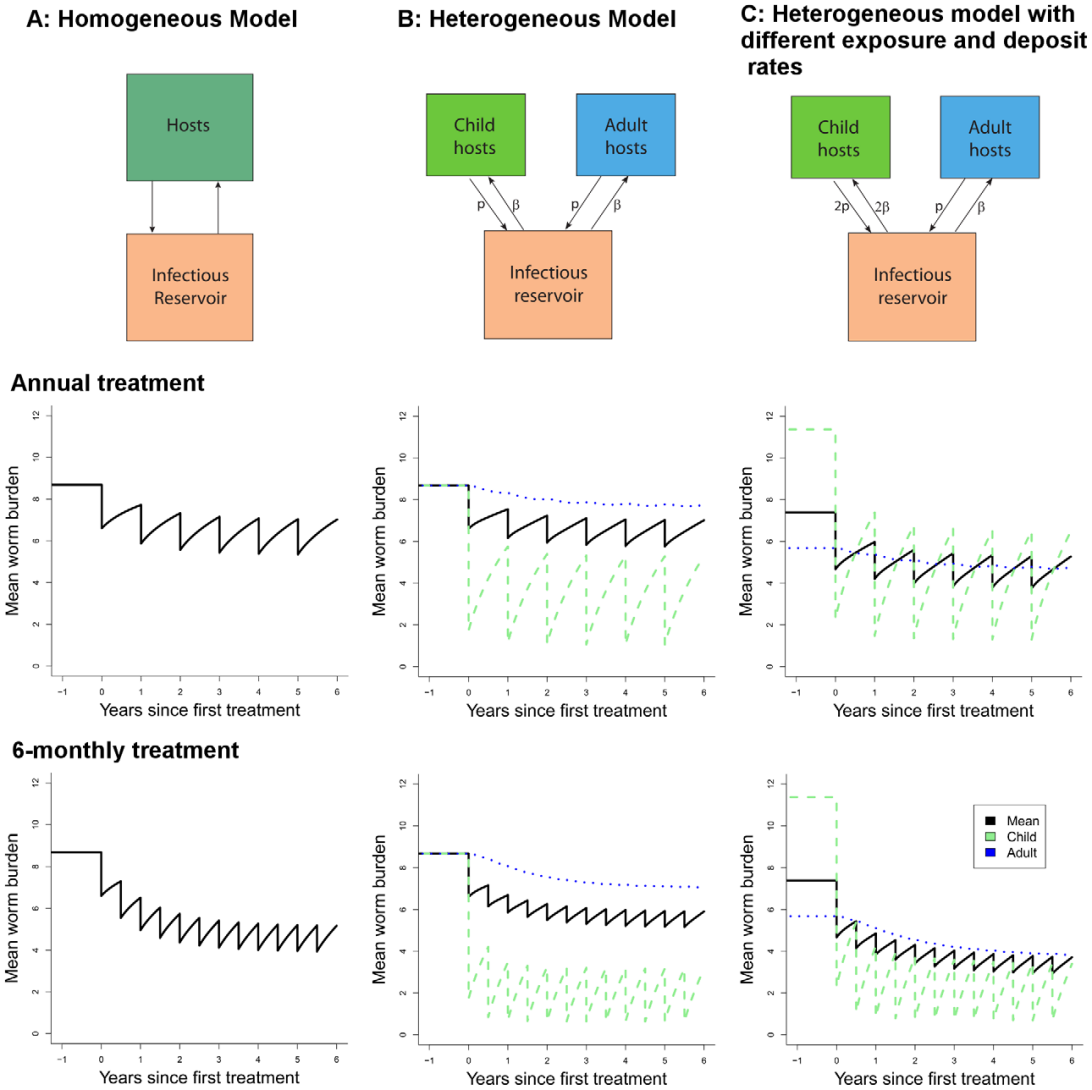
Effect of regular treatment on mean *A. lumbricoides* worm burden for different models. A homogeneous population (left column), B heterogeneous population with uniform transmission dynamics (central column) and C heterogeneous population with greater contribution from children (right column) as in the text. The two rows represent annual and half-yearly treatment respectively. For all runs, basic reproduction number is 3 and worm lifespan is 1 year. Other parameters (as defined for equations 6 and 7): *μ_2_* = 5/yr, *k* = 0.7, *z* = 0.93.

**Figure 8:**
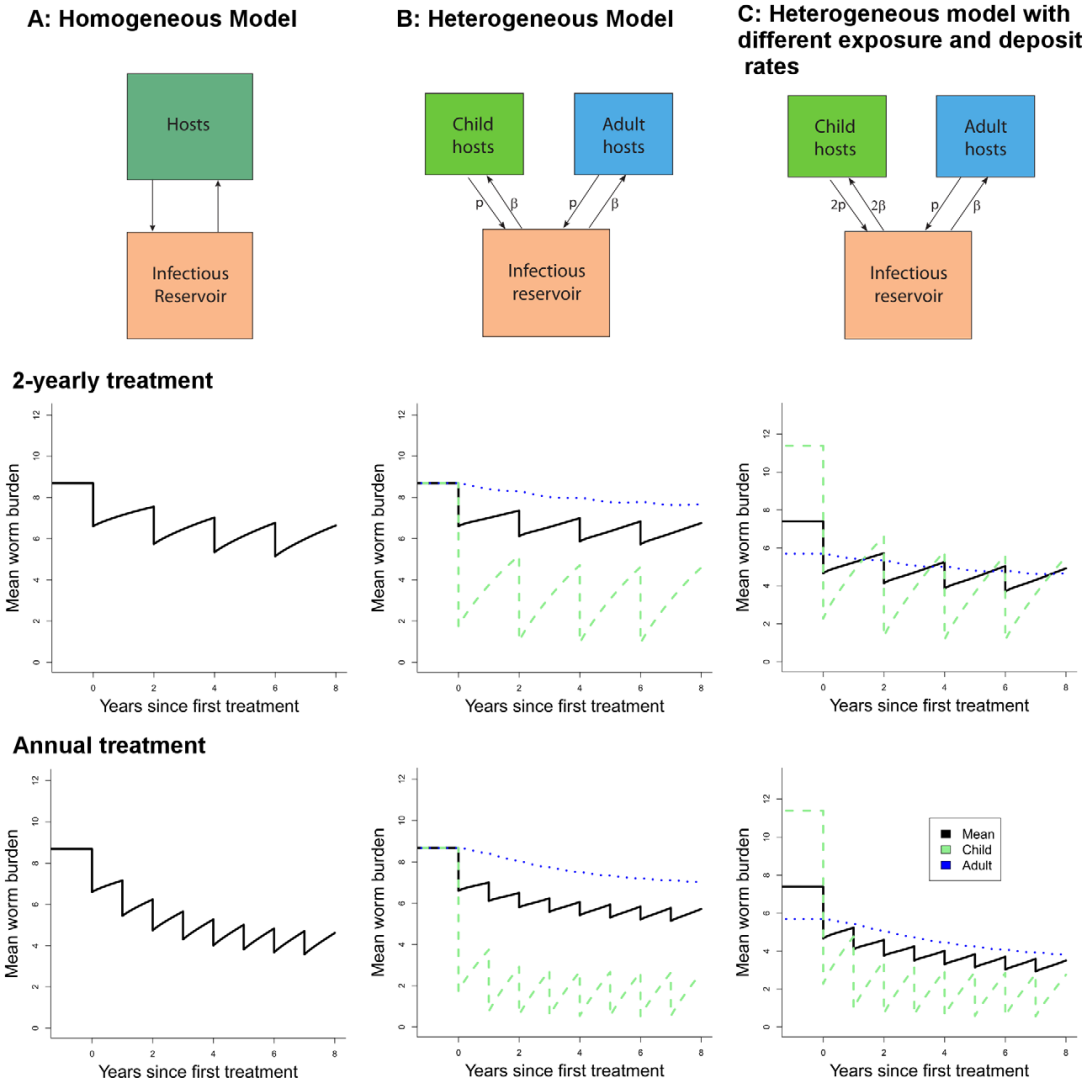
Effect of regular treatment on mean worm burden of hookworm for different models. As in Figure 7, the columns are A homogeneous model, B heterogeneous population with uniform transmission dynamics and C heterogeneous population with greater contribution from children, as in the text and different treatment intervals (rows). Simulations for basic reproduction number, 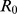 *= 3*, and worm lifespan is 2.5 years. Other parameters as in Figure 7. The two rows represent two-yearly and yearly treatment respectively.

Further insight on the effect of targeting school-aged children can be gained by considering differential mixing patterns between children and the rest of the population, as outlined above. The results of example simulations are presented in Figures 7 and 8, where the worm burdens in school-aged children and other age groups (where applicable) and averaged across the community are presented for different modelled scenarios, helminths and treatment intervals. The columns of the figures correspond to the scenarios A (homogeneous population), B (homogeneous mixing) and C (heterogeneous mixing). The heterogeneous mixing (scenario C) results in a higher worm burden in the children than in the adults, as is seen in several settings (note this model does not include any immunity). All models have the same mid-range 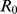 value of 3. Treatment in the homogeneous model is made comparable with the heterogeneous model by setting coverage to 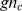. We have simulated these scenarios for *A. lumbricoides*, with a life expectancy of 1 year (Figure 7) and hookworm with a life expectancy of 2.5 years (Figure 8).

The most striking feature of Figure 7 and Figure 8 is the very modest impact of treatment of children on transmission, even at high levels of efficacy (95%) and coverage (85%). The treated children do have large benefits in terms of periods free of worms or with low worm burdens. However, the effect of treating children on worm burdens in the larger community is small. This reflects the proportion of the worm population actually reached by treatment, even though the chosen value (30%) is at the high end of school-attending fraction of the population (Table 5). Decreasing the treatment interval (bottom row in each figure) has only a moderate effect. The two group equal mixing model (scenario B) shows the direct effect of school-based treatment on school-aged children and also the indirect effect on adults through the reduction in infectious material in the community. The rate of bounce-back after treatment is slightly reduced in the heterogeneous model as compared to the homogeneous one (scenario B versus scenario A). This means that homogeneous descriptions (e.g. the homogeneous model, A) of non-uniform treatment regimens (targeted at some portion of the population) will always underestimate the time to recover to pre-treatment levels.

Scenario C mimics what we believe to be a more realistic epidemiological scenario, with school-aged children contributing twice as much infectious material as other age groups and also being twice as exposed, resulting in higher worm burdens in school-aged children. This is more likely to be observed for *A. lumbricoides* (e.g. Figure 3) than for hookworm (e.g. Figure 4). As such, the effect of treatment on the school-aged group is quite pronounced, but the impact at the community level, sometimes termed the ‘herd impact’ is only marginally improved, due again to the small proportion of worms treated. These simulations highlight the importance of mixing patterns in determining the effectiveness of school-based treatment programmes.

## Discussion

School-based approaches to deworming children have many advantages in terms of ease of access in urban and rural regions and the ability to link with other nutritional, health and education initiatives in order to try and minimize delivery and logistic costs. Advocacy for this approach to the control of STHs and the morbidity they induce has been made by many over the past decade [1], [30], [46]. However, with increased drug donations to support such programmes, it is now crucial to evaluate the benefits and disadvantages of such an approach. The limitations of this approach has already been implicitly acknowledged in the WHO recommendations to additionally target pre-school children, women of child-bearing age and high risk adults where possible [4] and previous identification of adults as a possible reservoir of infection [47]. However, there are many countries where only school-based deworming is currently under consideration [11]. Of particular importance in this context is the impact of school-based treatment on transmission of the parasites in the entire community, including the pre-school and adult age groups. In particular, sustained transmission (and thus production of infective stages) in other age groups will influence the frequency of treatment in school-aged children required to sustain infection at very low levels.

A limited number of field-based studies of mass chemotherapy have suggested that adult age groups who do not have access to treatment still benefit from school-based deworming as a result of its impact on the overall intensity of transmission within the population [30], [33], [48]. The treatment of a few (heavily infected individuals) can impact on the effective reproductive number and therefore reduced exposure to infective stages in those untreated. This effect is analogous to the concept of herd immunity in community-based vaccination programmes where vaccination reduces transmission to those still susceptible to infection. In the context of worms and chemotherapy, the herd impact arises from the reduction in the output of infective stages in faeces that result in the contamination of the environment in which the community of all age groups lives. A similar indirect protection among untreated individuals is the impact upon older children and adults seen after age-targeted (1–10 year olds) large-scale treatment of azithromycin against trachoma [49].

We employed two approaches to examine the impact of school deworming programmes on infection within the entire community, the ‘herd impact’ of the treatment programme. The first was empirical and based on the calculation of the proportions of the population in the school attendance age groups, the fraction of these age groups who attend school and the fraction of the total worm population harboured by in these school-aged children. The results of a set of calculations are summarised in Table 5. It should be noted that the age-profile and population pyramids used here were not exactly matched, and therefore the calculations will not be precise for a particular point in time. It highlights the need for more recent data for these pathogens, as highlighted in recent articles [50]–[51]. In many of the low-income settings in which STHs are highly prevalent, the population pyramid is approximately exponential in shape, meaning that from 20–30% (Table 2, Figure 3A and Figure 4A) are of school age. However, for some STHs, such as *A. lumbricoides*, the highest intensity infections are in this age-group (Figure 3B) and therefore a large proportion of worms are targeted by school-aged treatment (up to 50% in our examples, Table 5). These predictions are similar to empirical findings by Bundy et al. [30] who evaluated the impact of age-target chemotherapy on community transmission on the island of Montserrat, West Indies, where *T. trichiura*, which has similar transmission dynamics to *A. lumbricoides*, was the predominant species and had a prevalence of 12% (*A. lumbricoides* and hookworm occurred at <2%). The authors found that 4-monthy treatment of 2–15 year olds had a subsidiary effect on intensity of untreated 16–25 year olds. In contrast to *A. lumbricoides* and *T. trichiura* which exhibit age-convexity in intensity, hookworm intensity tends to monotonically increase with age (e.g. Figure 4B), as has been seen in several studies [52]–[54], and therefore a smaller proportion of worms or egg output are targeted by school-aged treatment (<10% in one of our examples, Table 5).

The different age profiles for these helminth species are a result of differing behaviour patterns, force of infection, heterogeneous exposure and, arguably, immunity and genetic pre-disposition. The details of the biology which generate these patterns do not need to be understood for the calculation of the proportion of worms treated. However, to estimate the impact of treating children on transmission in the larger community we need a combination of additional studies and novel modelling analyses. Our simulations (Figures 7 and 8) show the importance of understanding the nature of the interaction between school-aged children and the rest of the community in order to optimise treatment programmes in the longer term. It is possible that school-aged deworming will give little benefit outside the children being treated and that intensifying either the treatment coverage within this age-group and/or increasing the frequency of treatment (bottom rows in Figures 7 and 8) may not lead to the desired benefits in the larger community. In this case, other strategies will be needed to increase the potential for long-term control and, if the breakpoint can be crossed by combinations of interventions, eventual elimination of these helminths.

**Figure 9:**
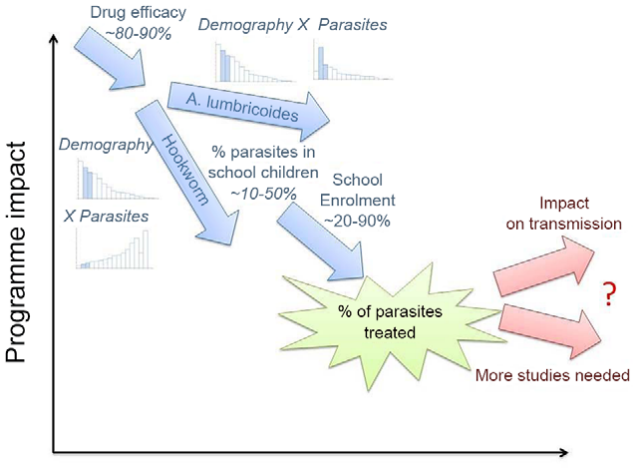
Schematic illustration of the impact of school-based deworming on the transmission of parasites. The number of parasites affected by a school-based deworming programme is never 100%, it is reduced by the efficacy of the drug, the proportion of the population of school age and their intensity of infection, as well as school enrolment and attendance on a deworming day. The impact of such a treatment programme on transmission is less easily quantified and depends on the details of transmission between different age-groups in the population. For further details, see main text.

As the schematic, Figure 9, shows, the impact of school-based treatment programmes on transmission in the larger community is diluted by a number of effects. The benefits of deworming for the affected children are many, but if we are to plan for long-term control and, in the longer term, elimination of these pathogens we need to consider strategies that will reduce transmission from year to year, as is already being discussed in some settings [55], particularly for schistosomiasis [56]. We also require an understanding of the species mix in each setting so as to tailor the design of interventions according to the underlying transmission dynamics. As this paper shows, there are many outstanding data gaps and needs for new modelling studies both to understand the dynamics of transmission under such programmes, and to design optimal treatment strategies for the future.
